# Ceftriaxone-Loaded Liposomal Nanoparticles for Pulmonary Delivery Against Lower Respiratory Tract Infections: Development and Characterization

**DOI:** 10.3390/ph18030414

**Published:** 2025-03-14

**Authors:** Vijay Kumar Panthi, Kathryn E. Fairfull-Smith, Timothy J. Wells, Tony Wang, Nazrul Islam

**Affiliations:** 1 Pharmacy Discipline, School of Clinical Sciences, Faculty of Health, Queensland University of Technology (QUT), Brisbane, QLD 4000, Australia; vijaykumar.panthi@hdr.qut.edu.au; 2 School of Chemistry and Physics, Faculty of Science, Queensland University of Technology (QUT), Brisbane, QLD 4000, Australia; k.fairfull-smith@qut.edu.au; 3 Centre for Materials Science, Queensland University of Technology (QUT), Brisbane, QLD 4000, Australia; 4 Frazer Institute, The University of Queensland, Brisbane, QLD 4102, Australia; 5 Australian Infectious Diseases Research Centre, The University of Queensland, Brisbane, QLD 4001, Australia; 6 Central Analytical Research Facility, Research Portfolio, Queensland University of Technology (QUT), Brisbane, QLD 4000, Australia; tony.wang@qut.edu.au; 7 Centre for Immunology and Infection Control (CIIC), Queensland University of Technology (QUT), Brisbane, QLD 4000, Australia

**Keywords:** ceftriaxone, liposome, nanoparticles, dry powder inhaler formulations, lower respiratory tract infections

## Abstract

**Background/Objectives:** Herein, we demonstrate the development and characterization of ceftriaxone (CTX)-loaded liposomal nanoparticles (NPs) intended to be applicable to the management of lower respiratory tract infections (LRTIs) associated with resistant bacteria. **Methods:** The CTX-loaded liposomal NPs were fabricated by a thin film hydration approach. **Results:** The particle size of the NPs, determined by a Zetasizer, was within the range of 90–536 nm. Microscopic examination by transmission electron microscopy (TEM) and scanning electron microscopy (SEM) revealed that particles are spherical in shape and have retained their original morphology even after freeze-drying. Attenuated total reflection-Fourier transform infrared (ATR-FTIR), differential scanning calorimetry (DSC), thermogravimetric (TG), and powder X-ray diffraction (PXRD) spectra exhibited that CTX is incorporated into the liposomes with no possible interaction between drug and excipients. The formation of the CTX-loaded liposomal NPs was dependent on the concentrations of phospholipids, cholesterol and mannitol; however, no considerable differences were observed in entrapment efficiency and loading capacity of CTX formulations (F6–F10). Using a twin-stage impinger (TSI), the in vitro aerosolization of the formulations were carried out at a flow rate of 60 ± 5 L/min and CTX was determined by a validated HPLC method and the prepared liposomal formulations produced promising fine particle fraction (FPF) between 47 and 62%. The prepared formulation (F6) showed prolonged CTX release of 94.0% ± 5.7 and 95.9% ± 3.9 at 24 h and 48 h, respectively. The drug release followed the Hixon–Crowell model, with CTX being transported through Fickian diffusion. **Conclusions:** These results highlight the prepared CTX-loaded inhaled liposomal formulation would be suitable for pulmonary delivery and extend the successful antibiotic delivery strategies for the effective management of LRTIs.

## 1. Introduction

Lower respiratory tract infections (LRTIs) are life-threatening lung diseases with serious health and economic impacts. LRTIs are often linked to lung-related illnesses like cystic fibrosis (CF), chronic obstructive pulmonary disease (COPD), and bronchiectasis. Numerous infectious gram-positive and gram-negative bacteria, including *Pseudomonas aeruginosa*, *Staphylococcus aureus*, *Streptococcus pneumoniae*, and *Burkholderia* species, are the primary causes of LRTIs [1,2], with prevalence estimates ranging from 10% to 50% [3]. Antibiotics, commonly administered orally or via injection to treat LRTIs, are inefficient to eradicate bacteria from infected lung cells. This study investigated the development and characterization of CTX-loaded liposomal NPs for pulmonary delivery.

Antibiotics are commonly administered orally or via injection to treat LRTIs. However, these methods have limitations, such as first-pass metabolism in oral administration and unsuitability for pediatric patients with parenteral routes [2]. Furthering this, the bioavailability of oral drugs is constrained by pre-systemic metabolism, leading to variability in absorption dynamics. Both oral and parenteral routes offer limited targeting capability that leads to inadequate therapeutic distribution at the target site [4]. Although parenteral delivery provides rapid and elevated drug levels in the bloodstream, it enhances the possibility of adverse reactions due to a narrow therapeutic window [5]. Studies [6,7] demonstrated that the systemic administration of antipseudomonal antibiotics reveals a likelihood of considerable toxicity for patients. For instance, administering high doses of intravenous (IV) tobramycin elevates the likelihood of systemic side effects, including nephrotoxicity and ototoxicity [6]. Additionally, IV use of broad-spectrum antibiotics can disturb the normal gut microbiota, heighten the possibility of secondary infections like *Clostridium difficile*, and contribute to the development of antibiotic resistance [7]. To overcome these challenges, studies have been shifted toward inhaled delivery of antimicrobial agents. This technology entails delivering the drug directly to the infected cells, achieving high local drug concentrations that effectively eliminate bacteria while minimizing side effects at a greatly reduced dose [8]. Inhaled liposomal antibiotic delivery has proven to be an efficient treatment option for the management of LRTIs [9]. Liposomes facilitate drug delivery to alveolar macrophages, but intravenously administered liposomes encounter difficulties such as premature release, quick elimination, and uptake by lung macrophages [10,11,12]. In contrast, inhaled drug-consisting liposomes can access deeper areas of the lungs, interact with alveolar surfaces, release antibiotics, and kill intracellular bacteria [13]. Liposomal nanoparticles (NPs) retain stability and do not aggregate when introduced to bronchoalveolar lavage fluid (BALF), and alveolar macrophages do not saturate their interaction with these liposomes [9]. For instance, an oral dose of ciprofloxacin, even at twice the maximum labelled dose of 750 mg, results in a peak drug concentration in sputum that is below the minimum inhibitory concentration (MIC) of 4 µg/mL for *P. aeruginosa* in biofilms [14]. In comparison, inhalation of ciprofloxacin liposomes achieves a peak sputum concentration exceeding the MIC by greater than 50-fold, with the average concentration over a 24 h treatment duration surpassing the MIC by 20-fold [15]. Additionally, liposomes offer promising compatibility with alveolar surfactants, which are primarily composed of lipids (around 90%) and play a vital role in lung activity [9].

The commercially available inhaled liposomal formulations of amikacin (trade name Arikayce™) and ciprofloxacin (rapid release: Lipoquin™ or ARD-3100, slow release: Pulmaquin™ or ARD-3150) have shown significant improvements in lung function compared to their non-liposomal counterparts in clinical studies of infected lungs [2]. Ceftriaxone (CTX) has shown effectiveness against multi-drug-resistant bacteria such as *P. aeruginosa* and *S. aureus*, which are associated with LRTIs [16].

Inhaled liposomal drug taken up by lung compartments, preferably by alveolar macrophages, showed extended drug action compared to the free drug [17]. CTX, effective against multi-drug-resistant bacteria [18], is only available as an injectable dosage form, which has dose-related off-target toxicity. Antibiotics with poor lung penetration, such as β-lactams and cephalosporins, are ineffective against multi-drug-resistant bacteria and are not beneficial for treating LRTIs [19]. To the best of our knowledge, CTX-encapsulated inhalable liposomal NPs have not been studied. Therefore, this study focused on developing and characterizing CTX-loaded liposomal NPs for pulmonary delivery. The targeted CTX delivery with possible site-specific extended antibiotic release by interacting liposomes with alveolar macrophages would help eradicate intracellular bacteria. This expected potential of inhalable CTX-loaded liposomal NPs for pulmonary delivery would extend the successful antibiotic delivery strategies for the effective management of LRTIs. The developed NPs were assessed based on their physicochemical properties, capacity of drug entrapment and loading, release profile, solid phase characteristics, as well as flow and aerosolization efficiency, with the goal of optimizing the dry powder inhaler (DPI) formulations for the potential future application against LRTIs.

## 2. Results and Discussion

### 2.1. Preparation and Rationale of Liposomal NPs

The various formulations of CTX-loaded liposomal NPs were fabricated by a thin film hydration approach. Altogether, ten liposomal formulations were developed, consisting of nine formulations of CTX-loaded liposomes and one with blank liposomes. All liposomal formulations were white-colored, with no phase separation and suspension-like consistency. Herein, the first formulation (F1) of CTX-loaded liposomes (Table 1) was prepared using 0.66%, 0.23%, and 0.82% *w*/*w* proportions of L-α-phosphatidylcholine, cholesterol, and Tween-80, respectively, where the former two excipients are the building components of liposomal NPs, and Tween-80 was added to enhance the liposomes flexibility and characterize various parameters of this formulation. In this study, 5 mg of CTX was used in all liposomal formulations, and the reason behind the use of this amount is that, as per the literature, CTX revealed MIC50 around 1 μg/mL against various pathogens, such as *S. aureus* and *Klebsiella pneumoniae* [20]. Thus, it is expected that the incorporation of 5 mg of CTX into liposomes will be enough to achieve desired antibacterial activity against several pathogens, including *P. aeruginosa*. As demonstrated in Table 2, F1 demonstrated that particle size within the nano range (286 nm), there was better drug entrapment (62.0%), and lower drug loading (5.0%). All these findings were satisfactory; however, the initial drug release from this formulation was quite slow and saturated at 12 h.

It is reported that to anticipate maximal therapeutic benefit against lung infections caused by pathogens, the initial burst and then sustained drug release is crucial [21]. To examine the impact of varying increased concentrations of phosphatidylcholine and cholesterol on drug release, the F2, F3, F4, and F5 were developed prior to enhancing the bilayer toughness of the liposomal membrane with a sustained release profile. Among all formulations, F3 revealed excellent entrapment and better loading efficiency (Table 3) with the desired release pattern (initial burst and sustained). This formulation resulted in bursts and prolonged drug release. After examining all these parameters, blank and CTX-loaded freeze-dried liposomal particles were analyzed to determine the powder density and flow property. However, the freeze-dried powdered form of all liposomal formulations was sticky, and the formulations did not reveal satisfactory flow, which might be due to the stickiness caused by the lipids (cholesterol, phosphatidylcholine, and Tween-80) used to prepare the liposomes and the highly hygroscopic property of CTX. Thus, all those formulations (F1, F2, F3, F4, and F5) were discontinued for determining the powder flow and in vitro aerosolization. To overcome this problem and prior to achieving the desired drug concentration for in vitro aerosolization efficiency, new formulations (F6–F10) were developed using a higher concentration of D-mannitol (750 mg) and L-leucine (50 mg) to minimize the hygroscopicity of CTX with the non-hygroscopic characteristic of mannitol due to its high crystallinity [22] and then characterized thoroughly. While this formulation includes phospholipids and an edge activator (Tween-80) and involves thin-film hydration followed by sonication, it differs from transferosomes overall, as it does not involve the freezing and thawing of the suspension. Additionally, edge activators are the second key component in transferosomes, typically comprising 10–25% of the formulation. However, in this study, the liposomal formulations contain only 2.5% edge activator [23]. Moreover, according to the literature, the reverse-phase evaporation method is well suited for high-molecular-weight molecules, such as peptides. In contrast, the thin-film hydration method involves dissolving all lipids and hydrophobic drugs in an appropriate organic solvent using a round-bottom flask. The hydration solution can include hydrophilic drugs intended for loading into the aqueous core of the liposomes. Therefore, in this study, CTX, a hydrophilic drug, was dissolved in the hydration solution before being loaded into the liposome core to achieve sustained release [24].

### 2.2. CTX Analysis by Reversed-Phase High-Performance Liquid Chromatography (RP-HPLC) and Method Optimization

In this research, CTX peak was effectively separated and eluted without tailing, showing a retention time of 1.7 min. The HPLC method for CTX was authenticated based on ICH standards, demonstrating linearity in the concentration range of 25–250 µg/mL, with an r^2^ of 0.9992. This high r^2^ suggests minimal random error and strong linearity, confirming a good correlation between the peak area versus CTX concentration data to the linear equation. The regression equation derived was y = 40.561x − 263.26, where *y* represents the peak area, and *x* stands for the drug concentration. The method’s efficiency is highlighted by the small injection volume of 20 µL and a run time of 7 min, enabling speedy analysis in standard laboratory procedures. The method’s selectivity was verified when no baseline deviations were observed after injecting an analytical placebo comprising all ingredients except CTX. The percentage recovery of CTX ranged from 98.61% to 101.56%; intra-day and inter-day precision, measured by % relative standard deviation (RSD), were between 0.89% and 1.83% (below 2%). The recovery percentages and % RSD values were within the acceptable limits of 98.0% to 102.0% and did not exceed 2.0%, demonstrating the method’s suitability for consistent drug examination [25,26]. Moreover, the limit of detection (LOD) and limit of quantification of CTX were 4.27 µg/mL and 6.45 µg/mL, respectively.

Several trials were conducted to optimize the mobile phase ratio and enhance chromatographic separation using a C18 (250 ×  4.6 mm, 5 µm) column. Based on the solubility behavior of CTX, the composition of the mobile phase was optimized. In the first trial, a Varian column and a diode array detector (DAD) set at 270 nm were used, with a mobile phase of ACN and water at a 70:30 *v*/*v* ratio and a 20 µL injection volume without pH adjustment. This resulted in a narrow, asymmetrical HPLC peak, leading to the trial’s rejection. The second trial maintained the same mobile phase composition but adjusted the pH to 6.5 using 0.01% triethylamine buffer, though the peak did not improve. In the third trial, a Phenomenex C18 column (250 × 4.6 mm, 5 µm) was tried with the same conditions as trial 2, resulting in a symmetrical but broader peak, so this trial was also rejected. Finally, in the fourth trial, a Varian column was used again, with the mobile phase modified to a 55:45% *v*/*v* ratio of ACN and water, producing a well-defined peak. This composition was accepted as the final mobile phase. The HPLC chromatograms of pure CTX, blank liposome, and CTX-loaded liposome are demonstrated in Supplementary Figure S1.

### 2.3. Particle Size, Polydispersity Index (PDI), and Zeta Potential Evaluation

The particle size, PDI, and zeta potential of blank and CTX-encapsulated liposomal NPs were evaluated before and after freeze-drying samples. In this research, as depicted in Table 2, freeze-dried samples demonstrated reduced particle size in all formulations when compared with the samples that had not been freeze-dried. It has also been reported that liposome size is sometimes considerably reduced after freeze-drying, revealing the presence of shrinkage induced by the lyophilization process [27]. However, PDI decreased only in a few formulations (F4 and F5). Particle sizes were in the range of 90–501 nm and 75–536 nm for before and after freeze-drying, respectively. This pattern was similar to the previous research performed by Wieber et al. [28], where they mentioned that the particle size of liposomes decreased markedly after freeze-drying [28]. Among all formulations, F2 and F3 revealed almost the same particle size, while PDI was almost 2.0-fold higher in F2 (0.444 ± 0.110) compared to F3 (0.245 ± 0.009). In addition, with a 2.0-fold increase in the phosphatidylcholine concentration (F2) compared to F1, liposome size was reduced significantly, and further enhanced the phosphatidylcholine concentration to four times, which was also less than F1; however, it did not decrease more than F2. The corresponding values of particle size for F1, F2, and F3 were 286.70 nm ± 52.57, 90.66 nm ± 8.16, and 93.33 ± 0.45, respectively. This finding aligns with a study performed by Linda and team [29], where a higher amount of phospholipid resulted in smaller particle sizes upon high-pressure homogenization. F3 exhibited less PDI, and it can be stated that smaller particle sizes resulted in improved PDI [30]. The particle size distribution curve of blank and drug-loaded liposome (F3) is shown in Supplementary Figure S2. Moreover, elevating the cholesterol concentration by 2-fold (F4) than F1 led to particle size enhancement, and a further increase (4-fold) in the cholesterol concentration (F5) also resulted in a larger particle size; however, no noticeable variation was observed in zeta potential. Thus, in this study, formulations prepared with a higher amount of cholesterol were greater in size than other formulations. This finding is similar to previous research performed by Khan and team [31]. It has been observed that adding cholesterol can increase the hydrophobicity of particle surfaces. This heightened surface hydrophobicity may lead to particle aggregation, which in turn can result in an increase in particle size [31]. The average particle size, PDI, and zeta potential of liposomes before and after freeze-drying were shown in Table 2.

In this study, all liposomal NPs exhibited a negative charge (approximately −45 to −60 mV, Table 2), which is linked to the preferential uptake of OH- ions from water by the lipid particles [32], a phenomenon highlighted by numerous researchers [33,34,35]. Compared to F1, both F2 and F3 revealed lower negative values of zeta potential, indicating that proportionally increasing the concentration of phosphatidylcholine led to a lesser negative zeta potential. This is likely because phosphatidylcholine also acts as a surfactant. It was confirmed that raising the phosphatidylcholine concentration enhanced the cationic nature of the formulation, thereby increasing the zeta potential [36]. Further to this, the drop in negative zeta potential values with increased phospholipid concentration may be attributed to the greater presence of phosphatidylcholine moieties on the liposome surfaces [37]. Furthermore, the higher cholesterol concentration in F4 and F5 resulted in more negative zeta potential compared to F1. This increased negative charge is owing to the uneven distribution of polarity in the cholesterol’s hydroxyl group [30]. On the other hand, after freeze-drying, all liposomal formulations demonstrated decreased zeta potential values other than F2. The decrease in zeta potential after freeze-drying is likely a consequence of the crystalline reconfiguration of fat crystals, which may alter the surface properties. Additionally, the transition of crystals to a stable form of saturated fatty glycerides may occur. This transformation modifies the surface ratio of crystals with diverse charges, resulting in an alteration in the measured zeta potential [32].

As revealed in Table 2, after freeze-drying, the formulations consisted of L-leucine, and a higher concentration of D-mannitol of all drug-loaded liposomes (except F8) revealed the higher particle size compared to the blank liposomes, which might be due to either the blank liposomes shrinking after freeze-drying or an elevation in particle size with the addition of CTX that could be associated with a reduction in the volume of hydration medium when the drug is loaded. Consequently, the surface area of the vesicles decreases, causing the nanocarrier particles to enhance in size to compensate for the reduced availability of water [38,39]. Freeze-drying of drug-consisted liposomes showed greater average particle diameter than fresh liposomes, likely owing to the disruption of hydrogen bonds among water molecules and phospholipid head groups, as noted in previous studies [40,41]. Initially, freeze-drying concentrates the liposomes as the ice front advances. As the water is removed, membranes are more likely to come into close contact, promoting fusion or aggregation of liposomes [42]. On the contrary, freeze-drying of F7 and F8 exhibited a very slight increase and decrease in particle size, respectively, which suggested that the elevated phosphatidylcholine concentration, combined with a cryoprotectant like mannitol, could efficiently safeguard the liposomes against membrane fusion [43]. The underlying mechanism could be associated with the even distribution of particles associated with the higher phosphatidylcholine concentration and the membrane-stabilizing capability of mannitol interacting with the polar head groups [30,44]. It has been reported that nanocarrier properties like size, shape, and surface functionality significantly impact lung drug deposition and bioavailability [45]. Particles smaller than 0.5 µm deposit in the alveoli via diffusion, while those 1–5 µm settle in the bronchioles through sedimentation. Larger particles (>5 µm) deposit via impaction, common in dry-powder formulations [45]. Surface charge influences mucosal penetration, with hydrophilic and anionic/neutral carriers being more effective. Lipid-based carriers are ideal for the inhaled delivery due to superior aerodynamics [45]. The developed liposomal NPs (0.1–0.5 µm, negative charge) would follow the diffusion mechanism, enhancing lung deposition due to increased particle mobility [46]. Furthermore, the PDI values in this study indicated that the liposomal nanoparticles were moderately monodisperse, neither highly monodisperse nor highly polydisperse, as the observed PDI values were not extremely low (<0.1) or high (>0.7) PDI [47].

### 2.4. Morphology Evaluation

**Figure 1:**
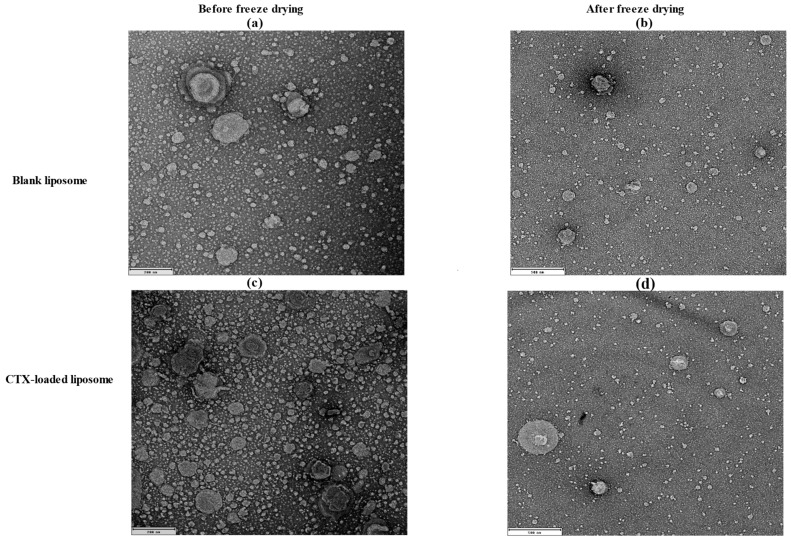
TEM images of liposomal NPs; (**a**) before (200 nm) and (**b**) after freeze-drying of blank liposomes (500 nm); (**c**) before (200 nm) and (**d**) after freeze-drying of drug-loaded liposomes (500 nm).

**Figure 2:**
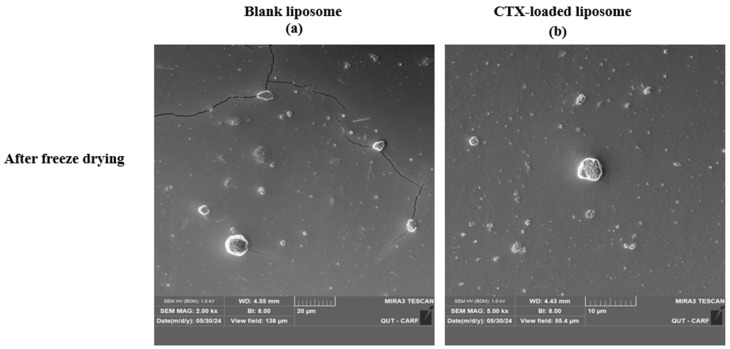
SEM images of freeze-dried liposomal NPs using; (**a**), blank liposomes (Magnification 2.00 kx), and (**b**) drug-loaded liposomes (Magnification 5.00 kx).

The morphology of liposomes was examined using transmission electron microscopy (TEM; JEOL 1400, JEOL Ltd., Tokyo, Japan) and scanning electron microscopy (SEM; Tescan Mira3, Brno, Czech Republic), which can reveal their shape, size, and the spatial configuration of phospholipids. Ideally, a liposome should be small, spherical, and free from roughness or rupture. Figure 1 displays TEM diagrams of liposomes made with or without drug loading formulations. In this study, both blank and CTX-loaded liposomes (F3) showed no irregularities in the vesicle membrane before and after freeze-drying. There was no aggregation, and the size distribution remained homogeneous with a smooth surface, indicating optimal arrangement of phospholipid molecules on the liposome surfaces. Additionally, TEM showed that both blank and drug-loaded liposomes were not fused even after freeze-drying. SEM was used to further confirm that liposomes are not fused after freeze-drying, and as depicted in Figure 2, both blank and CTX-encapsulated liposomes were not fused after freeze-drying.

### 2.5. Drug Entrapment and Drug Loading Determination

Drug entrapment and loading % were calculated for all CTX-loaded liposomal NPs using HPLC. The calibration curve of CTX standards was linear in the concentration range of 25 µg/mL–500 µg/mL. In this study, each liposomal formulation was prepared with the same quantity of CTX (5 mg). When the phospholipid concentration was doubled (F2 compared to F1), drug entrapment remained almost the same (Table 3), and when the phospholipid concentration was increased to 4-fold, entrapment efficiency decreased to 2.42%. An increase in phospholipid content in liposome formulation is reported to reduce entrapment efficiency [48,49]. This occurs because the higher phospholipid concentration leads to a larger and more fluid bilayer, creating additional space for molecules to move freely [48]. As a result, drug molecules are less effectively captured and retained within the liposome’s aqueous compartment, as the expanded membrane space allows for easy escape [48]. Similarly, a 2-fold enhancement in cholesterol concentration (F4 compared to F1) increased the CTX encapsulation into liposomes (6.04%); however, further enhancement in cholesterol concentration led to a decrease in drug entrapment by almost 2-fold (3.12%). This observation was also aligned with previous studies [37,50]. Generally, adding cholesterol or negatively charged phospholipid head groups helps stabilize membranes and liposomes, potentially leading to improved encapsulation efficacy [51]. However, Briguila et al. [52] observed that increasing cholesterol levels can decrease encapsulation efficiency. This occurs because, while cholesterol does not raise the transition temperature, it does increase membrane fluidity even below this temperature. This results in an elevated bending modulus (or bilayer toughness), which presumably explains the reduced encapsulation efficiency [53]. Cholesterol alters membrane properties and likely slows membrane fusion dynamics [54]. Previous studies have also reported minimal CTX entrapment in liposomes, with Shafiee et al. [55] documenting an entrapment efficiency of 1.16% and Diogo’s [56] research reporting 6%. On the contrary, among all liposome formulations, F4 demonstrated the highest drug loading, 2.00% ± 0.2, followed by F1 (1.54% ± 0.1), and F2 (1.43% ± 0.1). In this study, F9 and F10 showed the lowest drug loading capacity, which was 5.0-fold lower than F4. In addition, compared to F1, a 2-fold increase in cholesterol concentration in F4 led to enhanced loading capacity; however, a 4.0-fold increase in cholesterol concentration led to a reduction in drug loading capacity. Higher cholesterol levels in liposomes might reduce drug loading by stiffening the lipid bilayer, lowering permeability, and obstructing drug incorporation into the aqueous core. This effect is more significant for hydrophilic drugs, which require more space within the bilayer [57].

Increasing the D-mannitol and L-leucine concentration (F6–F10), the drug loading capacity was obtained with no considerable differences in all liposomal formulations. However, drug entrapment efficiency decreased. The F6 revealed 2.73% lower drug entrapment than F1. Entrapment efficiency is influenced by the porous nature of the carrier [58]. Due to D-mannitol’s crystalline structure and lack of porosity, the lipid tends to accumulate on the surface before liposome formation. Consequently, some lipid was lost as it adhered to the internal wall of the beaker and paddle stirrer. This reduction in lipid content in formulations with higher D-mannitol concentrations leads to less lipid being available for drug entrapment, ultimately resulting in lower entrapment efficiencies [58]. This result contrasts with the previous study in which authors reported that a higher concentration of mannitol opens the aqueous pores of liposomes that lead to higher entrapment for water-soluble drugs compared to a lower mannitol concentration [59]. F8 demonstrated maximal drug loading with no further improvement compared to F3 and a very slight alteration in F9 and F10, which might be due to the earlier bilayer toughness caused by higher phospholipid and cholesterol concentrations [50]. The overall findings of drug entrapment and loading efficiency for modified formulations are depicted in Table 3.

### 2.6. Attenuated Total Reflection-Fourier Transform Infrared (ATR-FTIR)

**Figure 3:**
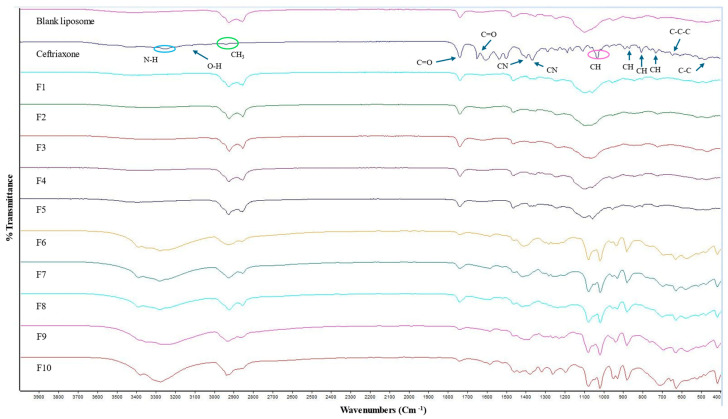
ATR-FTIR spectra of blank, CTX, and various CTX-loaded liposomal formulations.

The ATR-FTIR spectra for the blank liposome, CTX, and all other CTX-loaded formulations are displayed in Figure 3. The IR spectrum of plain CTX illustrates characteristic bands at 3150–3247 cm^−1^, reflecting the stretching vibrations of NH and OH groups. The absorption peak at 2936 cm^−1^ is related to asymmetric stretching of the CH_3_ group. Additionally, the stretching vibrations of C=O are identified at 1740 cm^−1^ and 1649 cm^−1^ and the peak at 1537 cm^−1^ is linked to the torsional vibrations of the aromatic ring. The bands at 1398 cm^−1^ and 1367 cm^−1^ are due to the stretching vibrations of the CN group [60,61]. The absorption peak at 1032 cm^−1^ is owing to the stretching vibration of =C-H [62]. Moreover, the CTX molecule contains three types of amides: amide I, II, and III. In the FTIR spectrum, the band ranges between 1610 and 1700 cm^−1^ and between 1480 and 1600 cm^−1^, and amide III 1220–1330 cm^−1^ are associated with amide I, amide II, and amide III, respectively [61]. The characteristic peak between 1610 and 1700 cm^−1^ ensures no interaction with other components [61]. C-H deformation vibrations are indicated by the weak bands detected at 822, 804, and 730 cm^−1^, which are assigned to out-of-plane deformation vibrations [63,64]. The very weak band at 507 cm^−1^ corresponded to C-C out-of-plane bending vibrations. Additionally, the weak bands at 646 cm^−1^ and 606 cm^−1^ are attributed to C-C-C in-plane and out-of-plane distortion vibrations, respectively [64]. The comparison of the FTIR spectra of blank liposomal NPs and drug-loaded liposomal NPs is the same and no characteristic peak of the drug is obtained in CTX-loaded liposomes, which indicates that drug is concealed in the liposomes or covered by other excipients used in formulations, and no interactions were observed between the drug and the excipients. Similar observations were also noted by previously published studies [65,66,67].

ATR-FTIR peaks were also not obtained in CTX-loaded liposomes consisting of a higher D-mannitol concentration and L-leucine, which is a result of the CTX being successfully entrapped in the liposomes or covered by other excipients used in formulations, and no interactions were observed between the drug and the excipients. Similar results were also demonstrated by others [65,67]. The IR spectrum of D-mannitol showed a peak at 3400 cm^−1^ attributed to the O-H stretching vibration. Peaks at 2956 cm^−1^ and 2903 cm^−1^ correspond to C-H stretching vibrations in -CH and -CH_2_ groups, respectively [68]. The ATR-FTIR spectrum for all excipients and blank liposomes (which consist of higher D-mannitol and L-leucine concentrations) are presented in Supplementary Figure S3.

### 2.7. Differential Scanning Calorimetry and Thermogravimetric Analysis

**Figure 4:**
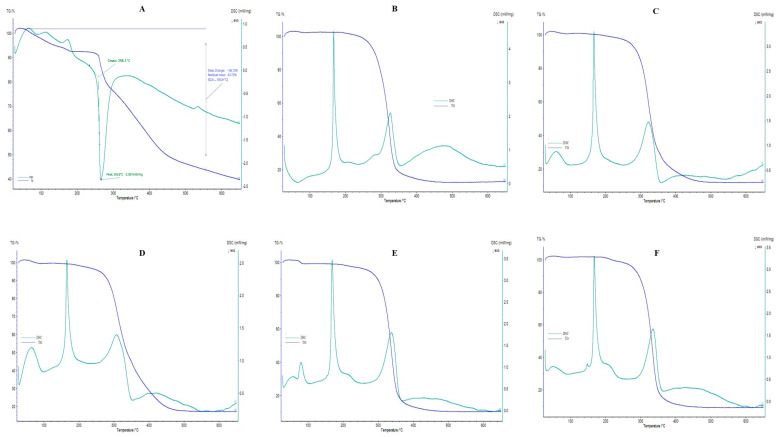
DSC/TGA thermograms. (**A**) CTX; (**B**) F6; (**C**) F7; (**D**) F8; (**E**) F9; (**F**) F10.

The DSC and TGA thermograms of pure CTX and F6–F10 are shown in Figure 4; blank liposomes and F1–F5 are presented in Supplementary Figure S4, and phosphatidylcholine, cholesterol, D-mannitol, and L-leucine are revealed in Supplementary Figure S5. In this research, a DSC thermogram of pure CTX revealed an endothermic peak at 75 °C, corresponding to the dehydration process, along with an exothermic peak at 265.6 °C related to the drug’s maximum decomposition. In addition, the DSC image of pure CTX showed an onset temperature at 258.1 °C, which indicates the thermal stability of CTX, where CTX starts to decompose initially. A similar observation was also reported by Abadi et al. [61]. The TGA thermograms represent gradual and successive weight-loss behavior. As the figure indicates, mass loss was obtained very slightly until at 250 °C, which might be attributed to the loss of water; however, after this temperature range, maximal mass loss appeared. To be specific, the temperature ranged between 52.6 °C and 555.9 °C, the 58.12% weight loss was obtained, and after 555.9 °C, and until 650 °C, the residual mass of pure CTX was 43.72%. In this study, CTX peak is not obtained in DSC thermograms of all drug-loaded liposomal formulations (F1–F10), which further confirms that the drug is successfully entrapped in liposomes which is similar to previously reported findings [67]. The peak detected at 152 °C in the DSC spectrum of cholesterol powder indicates the melting transition of cholesterol [69]. The DSC spectrum of D-mannitol powder illustrates an endothermic peak at 165 °C, exhibiting the melting of its crystal forms [70]. A broader peak around 350 C may result from polymorphic transformations, as D-mannitol is known for its unpredictable thermal behavior due to polymorphism [70]. Additionally, the DSC spectrum of L-leucine shows a peak around 300 °C, signifying its melting point [71].

### 2.8. Powder X-Ray Diffraction (PXRD) Analysis

#### Crystal Configuration of API Component Phases

Qualitative phase analyses of the measured XRD suggest that the five component phases used in this experiment are as follows: (1) ceftriaxone sodium hemiheptahydrate (PDF# 00-071-1637); (2) cholesterol (PDF# 02-061-1183); (3) D-mannitol (PDF# 02-069-8458); (4) L-leucine (PDF# 02-063-2332) [72]; and (5) amorphous phosphatidylcholine (Table 4).

**Figure 5:**
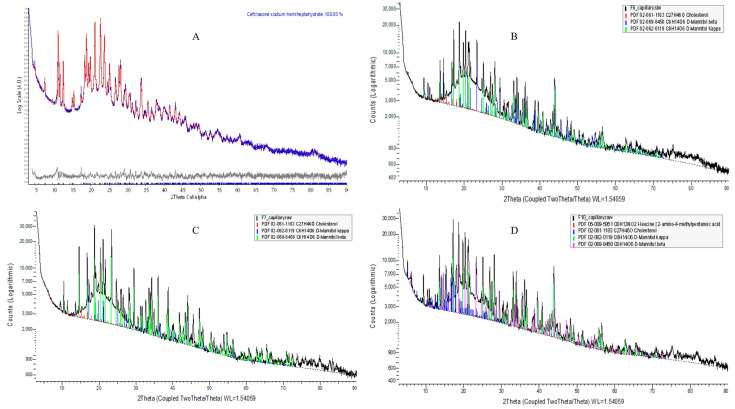
XRD patterns of pure CTX and CTX-loaded liposomal NPs. (**A**) Pure CTX; (**B**) F6; (**C**) F7; (**D**) F10.

L-α-Phosphatidylcholine is completely amorphous with no crystalline peak detected; however, cholesterol and D-mannitol (Supplementary Figure S6) and pure CTX (Figure 5A) revealed sharp crystalline peaks. The absence of distinctive CTX peaks in the prepared formulation suggests that the encapsulated drug is either dispersed at the molecular level or exists in an amorphous state within the liposomal NPs, aligning with the findings from DSC and ATR-FTIR analyses. In this study, D-mannitol’s form has been changed from beta to kappa after being incorporating into liposomes, which is due to either the concomitant crystallization behavior of mannitol polymorphs or the beta form of D-mannitol being reorganized to the kappa form after freeze-drying [73]. It has been reported that freeze-drying lysozyme solutions containing mannitol led to the formation of the β polymorph, along with some δ polymorph, but not the α form. This implies that the crystallization of mannitol polymorphs is impacted by the inclusion of additives [73]. The overall XRD peak images are demonstrated in Figure 5.

### 2.9. In Vitro Drug Release Study

**Figure 6:**
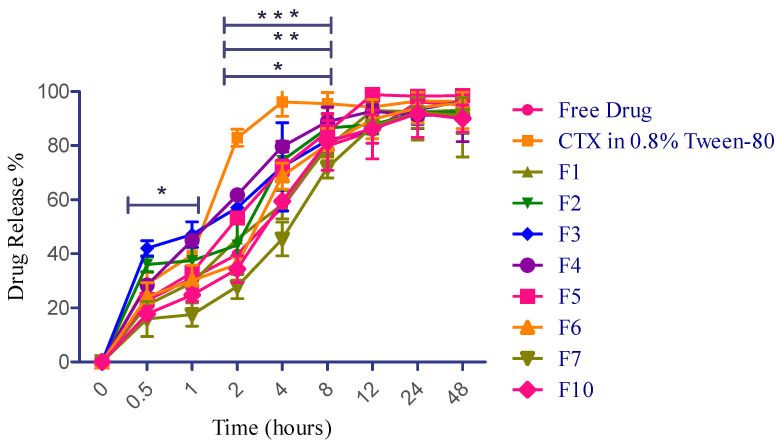
CTX release profile for free drug and CTX-loaded liposomal NPs. Data presented as mean ± SD (*n* = 3). * *p* < 0.05 at 0.5 h and 1 h for F3 vs. F7, CTX in 0.8% Tween-80 vs. F4 (2 h); F1 vs. F4 (4 h). ** *p* < 0.01 at 2 h for CTX in 0.8% Tween-80 vs. F3, CTX in 0.8% Tween-80 vs. F7 for 8 h. *** *p* < 0.0001 at 2 h for free drug vs. CTX in 0.8% Tween-80, F7 vs. CTX in 0.8% Tween-80, F10 vs. CTX in 0.8% Tween-80. CTX in 0.8% tween-80 vs. F1 (4 h).

The drug release of various formulations of CTX-encapsulated liposomes, free CTX solution (water), and CTX in water containing 0.8% Tween-80 was examined in a PBS (pH 7.4) using a dialysis bag. It has been reported that the initial burst and sustained release are expected to help achieve the desired drug concentration in the lungs. This would provide immediate and prolonged drug release, leading to optimal therapeutic benefits against LRTIs [21]. In this study, free CTX solution revealed sustained release throughout the study period, which is probably due to the ionization effect, as CTX (sodium salt hydrate) has a salt form. Weakly basic drugs dissolve well in acidic pH due to increased ionization in such environments [74]. However, another sample of CTX in water containing 0.8% Tween-80 remarkably modified the release pattern, which is attributed to the action of surfactant. This sample demonstrated an average drug release of 20.5% ± 0.9 in 15 min and 28.9% ± 4.3 in 30 min, respectively, and has a burst release of 82.8% at 2 h. This burst release value (82.0%) at 2 h is more statistically significant than free CTX solution and other liposomal formulations (F1, F2, and F5). Tween-80, as a surfactant, compromises the structural integrity of the dialysis membrane, facilitating the release of more drug [75]. Furthermore, liposomal preparations consisting of a higher concentration of phospholipid (F3) exhibited 42.0% ± 2.80 drug release at 0.5 h, followed by a prolonged release of 93.36% ± 5.39 and 96.41% ± 1.00 at 24 h and 48 h, respectively. Encapsulation effectively regulated the diffusion of CTX during its release. The initial minor burst might be attributed to minimal CTX adsorption and its presence in the outer region of liposomes. Following this, the CTX embedded in the core was released over an extended duration [76], as demonstrated in Figure 6. Additionally, in this study, F5 incorporated 2-fold higher cholesterol concentration than F4, wherein F5 revealed slightly more sustained release until 8 h; however, maximum drug release was obtained at 12 h for both F4 and F5. Overall, this research reported that liposomal formulations containing higher phospholipid concentrations resulted in a more sustained drug release up to 48 h, and a greater ratio of cholesterol-encapsulating formulations demonstrated controlled release up to 8 h.

Based on the better in vitro aerosolization efficiency (demonstrated in Section 2.12), altogether three best formulations (F6, F7, and F10) have been selected to further examine their drug release properties. In comparison with F1, the F6 fabrication very slightly helped to enhance the drug release at the earliest time periods, which might be due to the pore-forming action of mannitol within the dosage form [77]; however, it eventually extended the release saturation time from 12 h to 48 h. Liposomal preparations consisting of smaller concentrations of phospholipid and cholesterol with a higher mannitol concentration (F6) exhibited 24.1% ± 5.1 drug release at 0.5 h, followed by a prolonged release of 94.0% ± 5.7 and 95.9% ± 3.9 at 24 h and 48 h, respectively. On the contrary, F7 and F10 exhibited more sustained drug release than F2 and F5 from the initial duration but no statistical significance (*p* value: 0.8586) between these three formulations throughout the drug release period. The reason behind this finding is attributed to the bilayer rigidness caused by a higher concentration of phosphatidylcholine and cholesterol [53]. It has been reported that once drug particles are deposited in the lungs, they may be removed through various clearance mechanisms such as mucociliary clearance, macrophage phagocytosis, dissolution, and displacement from the airways to other areas. As a result, drug levels in the lungs can quickly decrease, potentially diminishing therapeutic effectiveness [78]. For instance, in bacterial lung infestations, the epithelial lining fluid (ELF) is particularly vulnerable to pathogens [79]. To attain optimal pharmacological effects and prevent resistance, it is crucial to maintain a specific amount of inhaled antibacterial drug in the ELF. If these agents are quickly absorbed into the systemic circulation or cleared from the lungs through several mechanisms, the ELF will not retain adequate antibacterial drug concentrations, reducing their effectiveness. In the worst-case scenario, this could lead to the development of significant antibiotic resistance. To ensure sufficient drug levels at the target areas, patients often need to take the medication frequently, leading to poor compliance and adherence. Prolonging the residence time of inhaled particles in the lungs is an efficient approach to obtain extended therapeutic effects [21]. Therefore, based on the release pattern observed in this study, the initial burst and sustained release of CTX (F3, F4, and F6) are expected to help achieve the desired drug concentration in the lungs from 5 mg of incorporated CTX to kill the bacteria-caused LRTIs. This would provide immediate and prolonged drug release, leading to optimal therapeutic benefits against LRTIs. The overall findings of drug release are depicted in Figure 6.

### 2.10. Kinetics of Drug Release

By applying various mathematical models to the in vitro release data and analyzing the release kinetics, this research aimed to elucidate the drug transport mechanisms through the liposomal membrane. This approach allows for quantitative predictions about how the CTX NPs will release the drug at the target site. The kinetic models applied to the data include zero-order, first-order, Higuchi, Hixon–Crowell, and Korsmeyer–Peppas models. The data interpretation was based on the r^2^ values, as revealed in Table 5. Based on the regression coefficients, it can be inferred that the Hixon–Crowell model best describes the release kinetics for most of the liposomal preparations (F1, F2, and F5), the control, and CTX in 0.8% Tween-80. The corresponding r^2^ values for F1, F2, and F5 were 0.9806, 0.9159, and 0.9253, respectively, indicating that diffusion is the dominant mechanism behind the release process. Additionally, compared to other liposomal formulations, F1 was also well fitted with the first-order and Higuchi models. The Hixon–Crowell model is applicable when drug release occurs from parallel planes on the surface of the dosage form, such as in tablets, where the size decreases proportionally while the shape remains constant. Similarly, the Higuchi equation relies on various assumptions: the initial drug concentration in the formulation exceeds the drug’s solubility, resulting in unidirectional drug diffusion; the drug particles are smaller than the carrier; system swelling and dissolution are minimal; drug diffusivity remains constant; and sink conditions are maintained [80]. In this study, the zero-order and Korsmeyer–Peppas models were not suitable for describing the release of CTX from liposomes (F2–F5), control, and CTX in 0.8% Tween-80. Furthermore, as per the Korsmeyer–Peppas model, drug transport follows Fickian diffusion when the parameter *n* is less than or equal to 0.5, while an *n* value between 0.5 and 1.0 suggests anomalous transport [80]. For all the samples in this study, the *n* values ranged from 0.1526 to 0.2383, illustrating that the release of CTX from the prepared fabrications was governed by a diffusion mechanism. The *n* value obtained in the control and Tween-80 solution also represented that CTX release was associated with the diffusion mechanism, and the corresponding *n* values for these samples were 0.2756 and 0.1947, respectively. This study affirmed that the drug release from the liposomal NPs was best described by the Hixon–Crowell model, with CTX being transported through Fickian diffusion.

Several mathematical models were applied to further assess the in vitro release data and analyze the release kinetics of liposomal formulations (F6, F7, and F10) consisting of L-leucine and a higher concentration of D-mannitol to examine the drug transport behaviors through the liposomal membrane. In the context of the r^2^ and *n* values, there is no considerable difference between F1, F2, and F5 compared to F6, F7, and F10. However, F7 followed all release kinetic models, revealing a higher r^2^ value (0.9963) followed by the Hixon–Crowell model, and owing to the *n* value obtained being less than 0.5 from the Korsmeyer–Peppas further highlighting that the CTX release from all fabrications was also controlled by a diffusion mechanism [80]. Table 5 demonstrates the release kinetics of CTX from all liposomal preparations.

### 2.11. Powder Density and Flow Property Evaluation

In general, the flow behavior of powders is assessed using the Carr’s index (CI) and the Hausner ratio (HR). A CI value below 25% signifies good flow, while a value above 25% suggests poor flowability, characteristic of cohesive powders. Similarly, an HR value lower than 1.25 signifies good flowability, while values above 1.25 illustrate poor flowability. However, these measurements provide only an estimate of the flow properties of formulated liposomal NPs, as the methodologies used for measurement can influence these values [1]. The CI values of freeze-dried liposome powders ranged between 16.25% and 22.10% and HR values from 1.19 to 1.26. All formulations revealed CI < 25%, indicating good powder flowability [81]. F6 and F8 also revealed < 1.25 HR values, demonstrating these formulations create better flow properties in both parameters. Although blank liposomes, F7, F9, and F10 exhibited HR values ranging between 1.25 and 1.28, these formulations can also be considered as having good flow properties as CI of all these formulations were less than CI < 25%. In this research, all modified liposomal formulations exhibited potential flow characteristics, which might be advantageous for the distribution characteristics of the liposome-based inhaled preparations.

The angle of repose (ϑ) values can also offer valuable insight into the flow properties of the formulated NPs. All formulations except F7 demonstrated ϑ values ranging from 36.49 to 38.54, revealing fair flow. F7 exhibited slightly poorer flow properties for all parameters (CI, HR, and ϑ) than other formulations, which might be due to the lesser particle size of this formulation (Table 6). It is reported that the particle size and its distribution are recognized as key factors influencing not only flowability but also parameters such as bulk density, ϑ, and compressibility of bulk solids. Even small changes in particle size can lead to remarkable shifts in flowability. Specifically, a decrease in particle size typically results in decreased flowability owing to an elevated surface area relative to mass [82]. Finer particles and a wider particle size dispersion both enhance cohesive forces and diminish flowability [83]. The smaller particle size enhances the contact area among particles, thereby amplifying cohesive forces [84].

### 2.12. Aerosolization Performance of the Formulations

The evaluation of in vitro aerosolization of all CTX-loaded liposomal NPs was performed with respect to recovered dose (RD), emitted dose (ED), fine particle fraction (FPF), and fine particle dose (FPD) and shown in Table 7. All fabrications showed RD values ranging from 88.4% to 92.8% (Table 7), indicating that the particles were effectively distributed from the mouthpiece and across different stages of the twin-stage impinger (TSI). However, particle surface charge might have caused them to adhere to the glass surface of the inhaler device, preventing the full dispersion of the remaining particles [1]. The ED of all liposomal formulations was between 59% and 69%. In this research, to compare all parameters of aerosolization efficiency of blank liposomal NPs with CTX-loaded liposomal NPs, the validated method of gravimetric estimation developed in our laboratory [1] was followed using filter paper (orifice 0.20 μm, Phenomenex, Torrance, CA, USA), and then filter washings from each stage of the TSI device were allowed to dry at 60 °C for 24 h. However, after drying, the filter paper did not obtain a constant weight. The reason behind this is that blank liposomal particles are completely filtered from 0.20 μm, as the particle size of the blank liposome was less (155.27 nm) than 0.20 microns. F9 revealed less RD-based FPF (31.97%) compared to other drug-loaded formulations, which might be due to the less negative charge of this formulation, as it may contribute to demonstrating a less repulsive force that led to a minimal FPF. Similarly, higher FPF (44.55%) based on RD obtained from F10 is also attributed to higher negative charge, which eventually leads to maximal repulsive force, which is also followed by F7 and F6 having an FPF of 41.17% and 40.87%, respectively. These findings are aligned with previously published studies [1]. On the contrary, although F8 revealed better powder flow properties and higher negative charge in comparison with other formulations, FPD was achieved less from this formulation. The reason behind this finding is either the drug is more deposited in S1 owing to the powder adherence in this stage from a higher phosphatidylcholine concentration or, theoretically, a smaller amount of the drug is available due to the higher weight of NPs in this formulation. It has been reported that increased adhesion of aerosol droplets to the internal walls of the aerosol device results in a delayed or slower transfer of the formulation to the device reservoir [31,85]. Considering the overall results, it is evident that the inclusion of additional mannitol and leucine not only solved the stickiness issue and improved flow but also decreased the drug–drug cohesion force and enhanced dispersibility, ultimately leading to an enhanced FPF (47.1–61.4%) of CTX. This is further augmented by adding leucine as a dispersion enhancer, helping to reduce internal particle resistance of the particles and enhance the powder flow characteristics and ensure a random distribution and uniformity within the mixtures (F6, F7, and F10) [86]. Among all formulations, the FPF % of F10 was significantly higher (*p* value 0.0126 and 0.0176) than F8 and F9, respectively.

The amount of CTX obtained in S2 of TSI was between 19.53 and 37.83 μg (from 59.5 to 92.6 μg of loaded CTX, Table 7) with fabricated liposomal NPs; therefore, it is expected that this amount of CTX would penetrate deep into the lungs and be absorbed. Additional in vitro antibacterial and antibiofilm investigations will also be carried out to examine the efficacy of CTX-encapsulated liposomal NPs against pathogens responsible for LRTIs and evaluate the mucus-permeating capacity of prepared formulations. Nevertheless, prospective explorations are necessary to analyze the safety and effectiveness of the prepared formulations, which are beyond the scope of this manuscript.

## 3. Materials and Methods

### 3.1. Materials

Ceftriaxone (sodium salt hydrate) was purchased from Sapphire Bioscience, Redfern, New South Wales (NSW), Australia. L-α-Phosphatidylcholine (purity: ≥99%; saturation: 100 mg/mL at room temperature in chloroform, ethanol, and hexane containing 3% ethanol) was purchased from Sigma-Aldrich Pty Ltd. (Castle Hill, NSW, Australia). Cholesterol and D-mannitol were supplied from Sigma-Aldrich, St. Louis, MO, USA. HPLC-grade acetonitrile and analytical-grade ethanol were obtained from Fisher Chemical (Pittsburgh, PA, USA) and Thermo Fisher Scientific (Waltham, MA, USA), respectively. Chloroform was purchased from Sigma-Aldrich, Bayswater, Victoria, Australia, and deionized double distilled water (Milli-Q water, ARIUM^®^MINI, Sartorius Lab Instruments GmbH & Co., Goettingen, Germany) was facilitated in our laboratory.

### 3.2. Preparation of CTX-Incorporated Liposomal NPs

CTX-loaded liposomal NPs were developed by applying a thin-film hydration approach with phosphatidylcholine, cholesterol, and organic solvents (chloroform and ethanol) as the lipid compartment and polysorbate-80 and mannitol as the hydrophilic compartment. In this study, several liposomal preparations were fabricated by the thin-film hydration approach, varying the composition of L-α-phosphatidylcholine, cholesterol, and polysorbate-80. Briefly, from the solution containing L-α-phosphatidylcholine, cholesterol, and organic solvents, only solvents were evaporated employing a rotary evaporator (Buchi Rotavapor R-300, Switzerland), and then a round-bottom flask (RBF) consisting of the dried thin film was kept in the fume hood overnight to remove traces of solvents. The next day the required amounts of CTX, mannitol, and surfactant (Tween-80) were dissolved in deionized water (5 mL) with slight stirring, and this solution was added into the RBF for initial hydration of the prepared thin films. Then deionized water (5 mL) was added, and the solution was sonicated for around 45 min and stirred using a paddle (plastic) stirrer at 800 rpm for 90 min, and then final samples were placed at 4 °C. Finally, liposomal suspension was kept at −20 °C to ensure that samples are frozen, which was freeze-dried (Freeze Dryer Alpha 1–4 LD plus, Los Angeles, CA, USA) at −55 °C for 72 h.

### 3.3. Analysis of CTX by RP-HPLC and Method Optimization

CTX was analyzed using RP-HPLC equipped on an Agilent 1100 HPLC system (Waldbronn, Germany) with an autosampler, a Varian C18 column (250 × 4.6 mm), and a Diode Array Detector (Atlas). The mobile phase consisted of a combination of acetonitrile (ACN) and deionized water in a 55:45% *v*/*v* ratio. An isocratic separation method was employed with 1 mL/min and 25 °C for flow rate and column temperature, respectively. A 20 µL injection volume was used, and the run time was 7 min. CTX had a retention time of 1.7 min and was detected at an absorption wavelength (λ_max_) of 270 nm. CTX standards were prepared by serial dilution in deionized water at concentrations between 25 and 250 µg/mL, were analyzed in triplicate (*n* = 3), and a calibration curve was generated by correlating the mean peak area against the concentration.

### 3.4. Characterization of Prepared Formulations

#### 3.4.1. Particle Size, Polydispersity Index, and Zeta Potential Evaluation

Determination of the particle size, polydispersity index (PDI), and zeta potential of the prepared liposomal formulations were performed on a Zetasizer Nano ZS (Malvern Instruments Ltd., Malvern, UK). All samples were analyzed three times at room temperature and at a 90° angle. Liposomal suspensions were diluted 100-fold with deionized water, and the parameters were assessed utilizing Zetasizer Nano software (Version 1.34.002).

#### 3.4.2. Morphological Evaluation

The particle shape and surface morphology of the prepared formulations were examined by both TEM and SEM. The morphology of before and after freeze-drying of both blank and drug-loaded liposomes were evaluated using TEM. For TEM, liposome samples were diluted 100-fold with deionized water, and a drop of sample was placed onto a carbon-coated copper grid for 5 min. Subsequently, the sample was stained negatively utilizing a 2% *w*/*v* uranyl acetate solution for 5 min and left to air dry at room temperature. Excess stains were eliminated with filter paper. Furthermore, for SEM, a drop of sample (prepared for TEM) was cast on a poly-L-lysine-coated glass cover slip and allowed air dry overnight and then mounted on an SEM stub and coated with a 4 nm Pt conductive coating.

#### 3.4.3. Drug Loading and Drug Entrapment Evaluation

A total of 1 mL of liposomal suspension was centrifuged at 17,500× *g* for 30 min at 25 °C to yield a pellet, and the supernatant was discarded. The pellets were resuspended in methanol (600 µL), vortexed vigorously, and deionized water (400 µL) was added to it, and further centrifugation was conducted at the same conditions to obtain a clear supernatant. Finally, the supernatant was examined using a validated HPLC method, and the entrapment efficiency was determined. The experiment was performed in triplicate. The drug entrapment and loading capacity were analyzed by applying the following formula:

(1) $$\mathrm{Drug loading}\left(\%\right)=\frac{\mathrm{Entrapped drug}}{\mathrm{nanoparticle weight}}\times 100$$

(2) $$\mathrm{Entrapment efficiency}\left(\%\right)=\frac{\mathrm{Drug encapsulated}}{\mathrm{Total drug}}\times 100$$

#### 3.4.4. ATR-FTIR Evaluation

The ATR-FTIR spectra of the phosphatidylcholine, cholesterol, CTX, Tween-80, D-mannitol, L-leucine, blank liposome, and all CTX-loaded formulations were recorded using an ATR-FTIR (Model: Alpha, Bogen, Germany) with the aid of OPUS software (Bruker Alpha-P FTIR, Version 7.0 or 7.5, Bremen, Germany). The scanning range was set between 400 and 4000 cm^−1^.

#### 3.4.5. DSC and TGA Analysis

The thermal behavior and decomposition of the pure CTX, phosphatidylcholine, cholesterol, D-mannitol, L-leucine, blank, and all CTX-loaded liposomal formulations were examined by differential scanning calorimetry (DSC) and thermogravimetric analysis (TGA) utilizing a simultaneous DSC/TGA thermal analyzer. The analysis was performed with a NETZSCH STA 449F3 (Selb, Germany), using Netzsch Proteus 80 software; version 8.0.3 which examines heat flow and mass loss through DSC and TGA, respectively. Samples (5–8 mg) were sealed in aluminum pans and examined from 30 °C to 650 °C at a heating rate of 10 °C/min, maintaining a nitrogen flow of 20 mL/min. Each sealed empty pan was used for reference and baseline correction.

#### 3.4.6. PXRD Evaluation

The measurement of all powder samples was performed in capillary (internal diameter 1.0 mm) transmission geometry utilizing a Rigaku Smart Lab X-ray diffractometer with a goniometer radius of 300 mm. A Goebel mirror with a CBO-E module was employed to focus the X-ray beam from a CuKα X-ray tube (λ = 1.54059 Å, 40 kV 40 mA), followed by a 15 mm slit to limit the length. A 1 mm incident slit was utilized to match the X-ray beam size with the capillary diameter. Soller slits of 2.5° were utilized on both the primary and secondary beam paths. A Hypix3000 detector, operating in 1D mode with a PSD aperture of 20 mm, captured the diffraction signals, positioned behind a 6.6 mm anti-scattering slit and a 12 mm receiving slit. The capillary samples were rotated at 15 rpm while XRD patterns were recorded over 3 to 90° 2θ at a 0.02° step size in 1 h. The XRD findings obtained were compared with the ICDD PDF-5+ database utilizing DIFFRAC.EVA v7.2 software. Rietveld structure refinements were performed using DIFFRAC.TOPAS v7.13 software.

#### 3.4.7. In Vitro CTX Release Evaluation

To assess the drug release, CTX-encapsulated liposomal formulations and free CTX solution (water) and CTX in water containing 0.8% Tween-80 were poured into a dialysis bag (with a molecular cutoff of 12,400 Da) and then stirred in a phosphate buffered saline (PBS; pH 7.4) medium (50 mL) using a magnetic bar at 150 rpm and at a steady temperature of 37 °C. Sample withdrawal was carried out at different time intervals, and an equivalent volume of fresh buffer was added prior to maintaining the sink condition, and then the amount of drug release was measured using HPLC.

#### 3.4.8. Kinetics of CTX Release from Liposomal NPs

The release kinetics of CTX from the liposomal NPs were analyzed by determining the correlation coefficient (r^2^) for different kinetic models, including zero-order, first-order, Higuchi, and Hixon–Crowell. Additionally, the Korsmeyer–Peppas model was applied to explain the release mechanism of CTX from the liposomal NP by assessing the release exponent “n”.

#### 3.4.9. Particle Density and Flow Property Evaluation

The particle density and flow characteristics of the powder were evaluated utilizing CI, HR, and θ as per the formulas provided. The bulk and tapped density of the freeze-dried liposomal powder were assessed by applying a graduated cylinder in a tapped density tester (ERW-SVM101202, ERWEKA, Langen, Germany). A specific amount of powder (500 ± 0.5 mg) was measured into a 5 mL graduated cylinder to measure the initial volume (V0), followed by 100 mechanical taps on the cylinder to determine the new volume (V1). Finally, CI and HR were estimated using the following formulas:

CI = 100[(plain volume − tapped volume)/plain volume]

HR = tapped density/bulk density

The θ is a pivotal parameter for examining the flow properties of particles. To measure this, 500 ± 0.5 mg of freeze-dried liposomal powder was slowly poured through a glass funnel with an 18 mm diameter and a 2 mm orifice. The distance from the funnel’s base to the orifice was 50 mm. The height (h) and diameter (d) of the cone formed by the powder were applied to calculate the θ applying the following formula:

θ = tan^−1^(2h/d)

#### 3.4.10. In Vitro Aerosolization Study

The aerosolization properties of the formulated liposomal particles were examined utilizing a TSI (Copley Scientific, Nottingham, UK) according to *British Pharmacopeia* guidelines [87,88]. A Breezhaler^®^ (Novartis Pharmaceuticals Pvt Ltd., Macquarie Park, NSW, Australia) was employed as the dry powder inhaler (DPI) device. Deionized water was added to the TSI, with 7 mL in stage 1 (S1) and 30 mL in stage 2 (S2). The airflow within the TSI was maintained at 60 L/min, governed by a vacuum pump (D-63150, Erweka, Heusenstamm, Germany) and measured with a calibrated digital flow meter (Fisher and Porter, Model 10A3567SAX, UK). Approximately 20 ± 0.5 mg of the liposomal powder was filled into size 3 hypromellose capsules (Vcaps^®^ Plus, Capsugel, Switzerland), and capsules were loaded into a Breezhaler^®^ DPI and then activated by piercing the capsule. Subsequently, the device was actuated by a vacuum pump for 5 s at a flow rate of 60 ± 5 L/min, dispersing the powder across several stages of the TSI device. This process was repeated five times for each fabrication (*n* = 5). After each measurement, all TSI stages were individually washed with deionized water, and stirring was performed at 150 rpm for 48 h to allow complete release of the drug, and then finally the amount of CTX was analyzed using the HPLC method.

The aerosolization efficiency of the developed fabrications was assessed by calculating the RD, ED, and FPF using the below-mentioned formula. RD represents the aggregate sum of particles retrieved from the inhaler, S1, and S2. ED refers to the portion of the RD that is dispensed from the inhaler into S1 and S2. FPF denotes the portion of RD that is accumulated in S2 of the TSI.

(3) $$ED=\frac{S1+S2}{RD}\times 100$$

(4) $$FPF=\frac{S2}{RD}\times 100$$

### 3.5. Statistical Analyses

Statistical evaluations were conducted using the 10.0.2 version of GraphPad Prism. A one-way analysis of variance (ANOVA) followed by a post-Tukey’s comparison test was employed to assess the data. *p* < 0.05 was considered to reveal statistical significance. The data are presented as a mean ± standard deviation.

## 4. Conclusions

This study presented a feasible and efficient approach for developing inhalable CTX-loaded liposomal formulations for pulmonary administration for the management of LRTIs. The liposomal NPs were developed by applying the thin-film hydration technique and thoroughly characterized. All formulations exhibited particle sizes between 90 and 536 nm. Morphological assessments via TEM and SEM confirmed that the liposomes were spherical and did not fuse after freeze-drying. ATR-FTIR, DSC, TGA, and PXRD analyses confirmed the successful incorporation of the drug into the liposomes without any interaction between the drug and other components. The formulation with less phospholipid and cholesterol in addition to L-leucine and a higher D-mannitol concentration (F6) demonstrated a high drug entrapment efficiency of 90.4% ± 8.7, a drug loading of 17.4% ± 2.3, and a sustained drug release of 95.9% ± 3.9 up to 48 h, with 94.0% ± 5.7 released at 24 h. Based on the r^2^ and *n* value, it is concluded that drug release from the liposomal NPs was best described by the Hixon–Crowell model, with CTX being transported through Fickian diffusion. Optimized mannitol and leucine concentrations improved the powder’s flow properties, resulting in an FPF ranging from 47.1% to 61.4%. This suggests that DPI formulations of CTX-incorporated liposomal NPs with promising aerosolization properties are potential options for lung delivery for the efficient management of LRTIs. However, further studies are warranted to assess their therapeutic effectiveness and safety for pulmonary delivery.

## Figures and Tables

**Table 1:** Formulation of various liposomal NPs.

			Change in Phospholipid Concentration		Change in Cholesterol Concentration		D-Mannitol (7.5%) and L-Leucine (1 mL of 5%)				
	Blank	F1	F2	F3	F4	F5	F6	F7	F8	F9	F10
Drug and Excipient	Amount (g)	Amount (g)	Amount (g)	Amount (g)	Amount (g)	Amount (g)	Amount (g)	Amount (g)	Amount (g)	Amount (g)	Amount (g)
Phosphatidylcholine (L-α)	0.20	0.20	0.40	0.80	0.20	0.20	0.20	0.40	0.80	0.20	0.20
Cholesterol	0.07	0.07	0.07	0.07	0.14	0.28	0.07	0.07	0.07	0.14	0.28
Chloroform	10.00	10.00	10.00	10.00	10.00	10.00	10.00	10.00	10.00	10.00	10.00
Ethanol	10.00	10.00	10.00	10.00	10.00	10.00	10.00	10.00	10.00	10.00	10.00
Ceftriaxone	0.00	0.005	0.005	0.005	0.005	0.005	0.005	0.005	0.005	0.005	0.005
Mannitol	0.0055	0.0055	0.0055	0.0055	0.0055	0.0055	0.7555	0.7555	0.7555	0.7555	0.7555
Tween-80	0.25	0.25	0.25	0.25	0.25	0.25	-	-	-	-	-
L-leucine	-	-	-	-	-	-	0.05	0.05	0.05	0.05	0.05
Deionized water (for hydration with drug and mannitol)	5.00	5.00	5.00	5.00	5.00	5.00	5.00	5.00	5.00	5.00	5.00
Deionized water (for final volume)	5.00	5.00	5.00	5.00	5.00	5.00	5.00	5.00	5.00	5.00	5.00
Total	30.53	30.53	30.73	31.13	30.60	30.74	31.08	31.28	31.68	31.15	31.29
Net weight of liposome (gm)	10.00	10.00	10.00	10.00	10.00	10.00	10.00	10.00	10.00	10.00	10.00

**Table 2:** Average particle size, PDI, and zeta potential of various liposome formulations.

Formulations	Particle Size (nm)		PDI		Zeta Potential (mV)	
	Before Freeze-Drying	After Freeze-Drying	Before Freeze-Drying	After Freeze-Drying	Before Freeze-Drying	After Freeze-Drying
Blank liposome	169.4 ± 18.0	146.1 ± 12.4	0.347 ± 0.0	0.590 ± 0.0	−50.1 ± 1.9	−50.8 ± 1.3
F1	286.7 ± 52.6	208.4 ± 10.4	0.444 ± 0.1	0.623 ± 0.0	−55.2 ± 3.0	−47.5 ± 1.6
F2	90.6 ± 8.1	85.2 ± 1.1	0.444 ± 0.1	0.477 ± 0.0	−51.6 ± 2.7	−54.7 ± 0.9
F3	93.3 ± 0.4	75.6 ± 6.0	0.245 ± 0.0	0.518 ± 0.0	−45.8 ± 0.1	−33.1 ± 7.0
F4	428.7 ± 49.0	249.5 ± 6.9	0.568 ± 0.1	0.400 ± 0.1	−59.5 ± 3.1	−50.0 ± 2.5
F5	501.3 ± 16.1	258.8 ± 4.5	0.647 ± 0.0	0.263 ± 0.0	−60.9 ± 3.2	−45.4 ± 0.6
F6	173.10 ± 10.7	235.73 ± 5.9	0.459 ± 0.1	0.478 ± 0.0	−54.87 ± 1.1	−46.90 ± 0.7
F7	144.87 ± 0.8	152.53 ± 1.0	0.365 ± 0.0	0.402 ± 0.0	−54.13 ± 1.8	−49.60 ± 0.3
F8	124.53 ± 1.6	102.30 ± 1.3	0.289 ± 0.0	0.255 ± 0.0	−53.40 ± 4.1	−59.23 ± 0.3
F9	290.37 ± 25.0	396.97 ± 15.2	0.521 ± 0.1	0.517 ± 0.1	−59.57 ± 1.0	−44.90 ± 1.0
F10	413.40 ± 28.5	536.90 ± 44.4	0.518 ± 0.0	0.549 ± 0.0	−64.43 ± 1.9	−52.93 ± 7.4

Data expressed as mean ± SD (*n* = 3).

**Table 3:** Average CTX entrapment and loading capacity of various liposomal formulations.

Formulations	Entrapment Efficiency, %	Loading Capacity, %
Blank liposome	-	-
F1	4.43 ± 0.4	1.54 ± 0.1
F2	4.35 ± 0.3	1.43 ± 0.1
F3	2.42 ± 0.2	0.83 ± 0.1
F4	6.04 ± 0.7	2.00 ± 0.2
F5	3.12 ± 0.2	1.05 ± 0.1
F6	1.70 ± 0.1	0.58 ± 0.1
F7	1.67 ± 0.4	0.50 ± 0.1
F8	1.74 ± 0.4	0.51 ± 0.1
F9	1.45 ± 0.5	0.41 ± 0.2
F10	1.56 ± 0.2	0.40 ± 0.1

Data presented as mean ± SD (*n* = 3).

**Table 4:** Crystallographic characteristics obtained from Rietveld refinement of XRD results.

Component Phases		Ceftriaxone Sodium Hemiheptahydrate	Cholesterol	D-Mannitol
Chemical Formula		C_18_H_23_N_8_Na_2_O_10.5_S_3_	C_27_H_46_O	C_6_H_14_O_6_
Space Group		C121	P1	P212121
Lattice		Monoclinic	Triclinic	Orthorhombic
Lattice Parameters	a (Å)	30.593(3)	14.165(1)	8.6792(1)
	b (Å)	4.7611(2)	34.231(5)	16.9006(1)
	c (Å)	18.587(1)	10.4699(6)	5.55051(6)
	α (°)	90	94.576(9)	90
	β (°)	90.460(5)	90.578(5)	90
	γ (°)	90	96.400(8)	90
Unit Cell Volume (Å^3^)		2707.3(3)	5027.9(8)	814.17(2)
Molecule Nos. in Cell Z		4	8	4

**Table 5:** In vitro release kinetics of CTX from liposomal NPs.

Formulations	Zero-Order	First-Order	Higuchi	Hixon–Crowell	Korsmeyer–Peppas	
	r^2^	r^2^	r^2^	r^2^	r^2^	*n*
Control	0.8686	0.7679	0.9817	0.9762	0.8545	0.2756
CTX in 0.8% Tween-80	0.8811	0.9864	0.9574	0.9688	0.7018	0.1947
F1	0.8809	0.9882	0.9935	0.9806	0.8651	0.1838
F2	0.8801	0.6354	0.7084	0.9159	0.7659	0.2004
F3	0.6574	0.8372	0.7247	0.8156	0.7287	0.1765
F4	0.7343	0.4670	0.6246	0.8806	0.7221	0.1788
F5	0.8157	0.6335	0.7396	0.9253	0.8412	0.2383
F6	0.8389	0.9736	0.9660	0.9403	0.8893	0.2606
F7	0.9579	0.9943	0.9879	0.9963	0.9082	0.3301
F10	0.8774	0.9723	0.9804	0.9495	0.8775	0.2782

**Table 6:** Powder density and flow property of various liposomal formulations.

Parameters	Blank Liposome	F6	F7	F8	F9	F10
Bulk density (g/mL)	0.11 ± 0.00	0.13 ± 0.00	0.17 ± 0.00	0.21 ± 0.01	0.11 ± 0.00	0.16 ± 0.00
Tapped density (g/mL)	0.140 ± 0.00	0.171 ± 0.00	0.219 ± 0.00	0.25 ± 0.01	0.14 ± 0.00	0.21 ± 0.01
CI (%)	20.03 ± 1.01	19.88 ± 1.01	22.10 ± 1.20	16.25 ± 2.18	21.13 ± 2.24	20.23 ± 2.53
HR	1.25 ± 0.01	1.24 ± 0.01	1.28 ± 0.02	1.19 ± 0.03	1.26 ± 0.03	1.25 ± 0.04
ϑ	36.49 ± 0.64	38.54 ± 2.50	41.29 ± 0.59	37.93 ± 0.64	37.74 ± 2.54	37.64 ± 0.67

Data presented as mean ± SD (*n* = 3).

**Table 7:** Average in vitro aerosolization findings of various liposome formulations.

Formulations	RD (%)	ED (%)	FPF (%) Based on ED	FPF (%) Based on RD	FPD (μg) Based on ED	FPD (μg) Based on RD
F6	88.4 ± 4.87	59.1 ± 7.3	61.46 ± 8.02	40.87 ± 4.57	56.88 ± 7.42	37.83 ± 4.23
F7	91.98 ± 3.52	69.54 ± 12.05	55.73 ± 12.29	41.17 ± 6.13	43.52 ± 9.60	32.15 ± 4.78
F8	92.74 ± 3.46	66.03 ± 10.62	47.17 ± 13.08	32.81 ± 7.33	28.07 ± 7.78	19.53 ± 4.36
F9	90.39 ± 1.35	61.20 ± 8.08	48.15 ± 12.13	31.97 ± 6.18	41.85 ± 10.55	27.78 ± 5.37
F10	90.05 ± 6.95	67.86 ± 2.44	58.72 ± 4.19	44.55 ± 5.89 *	45.51 ± 3.25	34.52 ± 4.57

Data expressed as mean ± SD (*n* = 5). * *p* < 0.05 for F10 vs. F8 and F10 vs. F9.

## Data Availability

The original contributions presented in this study are included in the article/Supplementary Material. Further inquiries can be directed to the corresponding author.

## Supplementary Materials

The following supporting information can be downloaded at: https://www.mdpi.com/article/10.3390/ph18030414/s1, Figure S1. HPLC chromatograms: (A) Pure CTX, (B) Blank liposome, (C) CTX liposome. Figure S2. Particle size distribution of liposomes before freeze drying. (A) Blank liposome and (B) CTX-loaded liposomes (F3). Figure S3. ATR-FTIR spectrum of various excipients. Figure S4. DSC/TGA thermograms. (A) Blank liposome, (B) F1, (C) F2, (D) F3, (E) F4, (F) F5. Figure S5. DSC/TGA thermograms. (A) Phosphatidylcholine, (B) Cholesterol, (C) D-mannitol, (D) L-leucine. Figure S6. XRD patterns of Phosphatidylcholine, Cholesterol, and D-mannitol.
