# Supplementary material for: Ceftriaxone-Loaded Liposomal Nanoparticles for Pulmonary Delivery Against Lower Respiratory Tract Infections: Development and Characterization
Source: Pharmaceuticals (Basel). 2025 Mar 14;18(3):414. doi: 10.3390/ph18030414 (PMC11945751; doi:10.3390/ph18030414)
Supplement: Supplementary file 1 [file pharmaceuticals-18-00414-s001.zip › pharmaceuticals-3459266-supplementary.pdf]

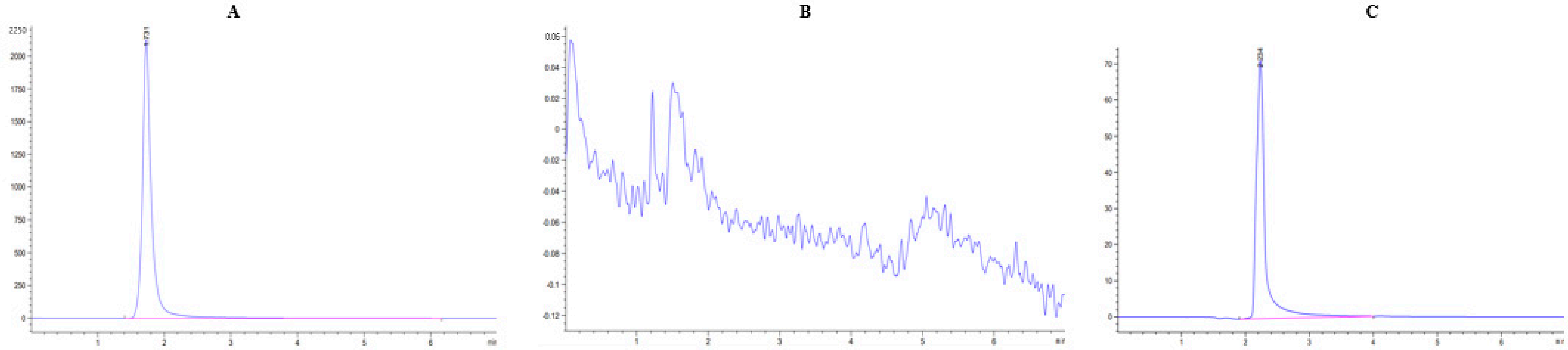

Figure S1. HPLC chromatograms: (**A**) Pure CTX, (**B**) Blank liposome, (**C**) CTX liposome.

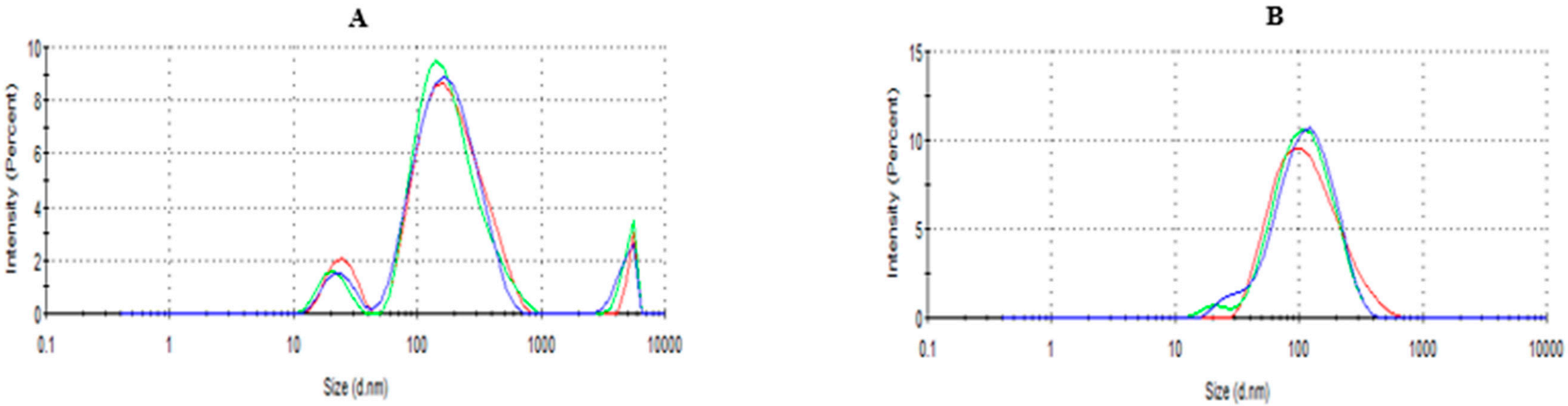

Figure S2. Particle size distribution of liposomes before freeze drying. (**A**) Blank liposome and (**B**) CTX-loaded liposomes (F3).

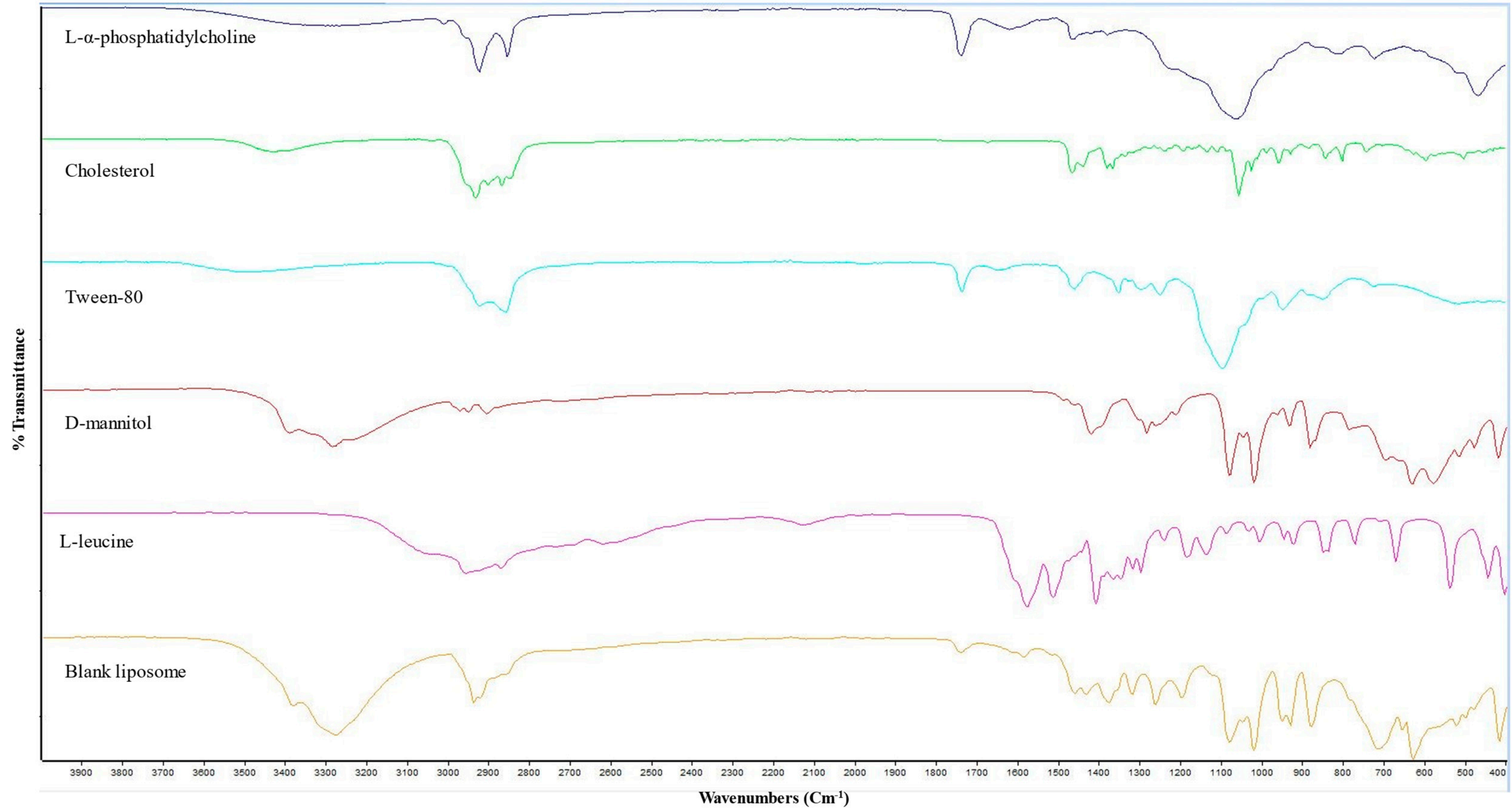

Figure S3. ATR-FTIR spectrum of various excipients.

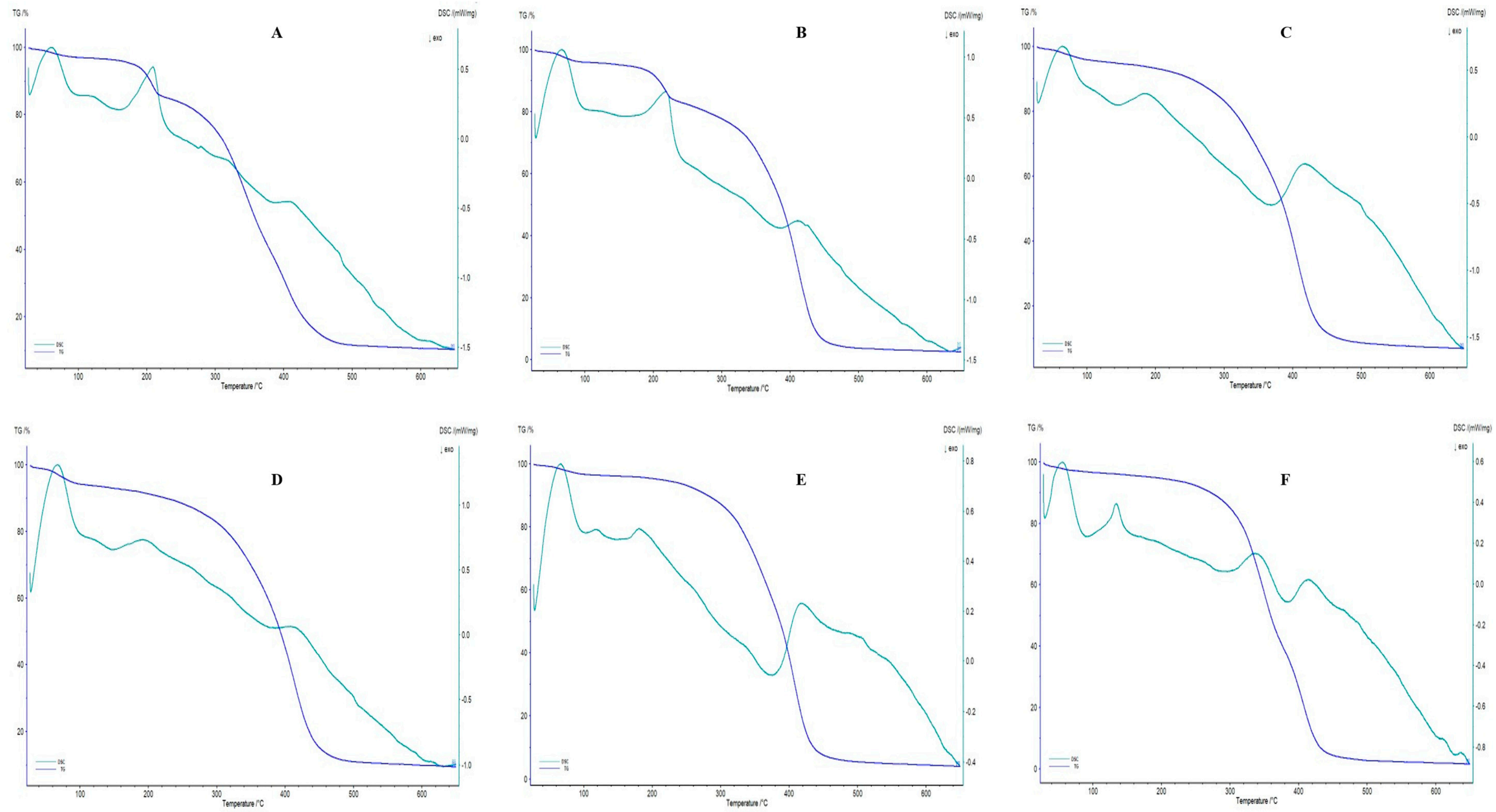

Figure S4. DSC/TGA thermograms. (**A**) Blank liposome, (**B**) F1, (**C**) F2, (**D**) F3, (**E**) F4, (**F**) F5.

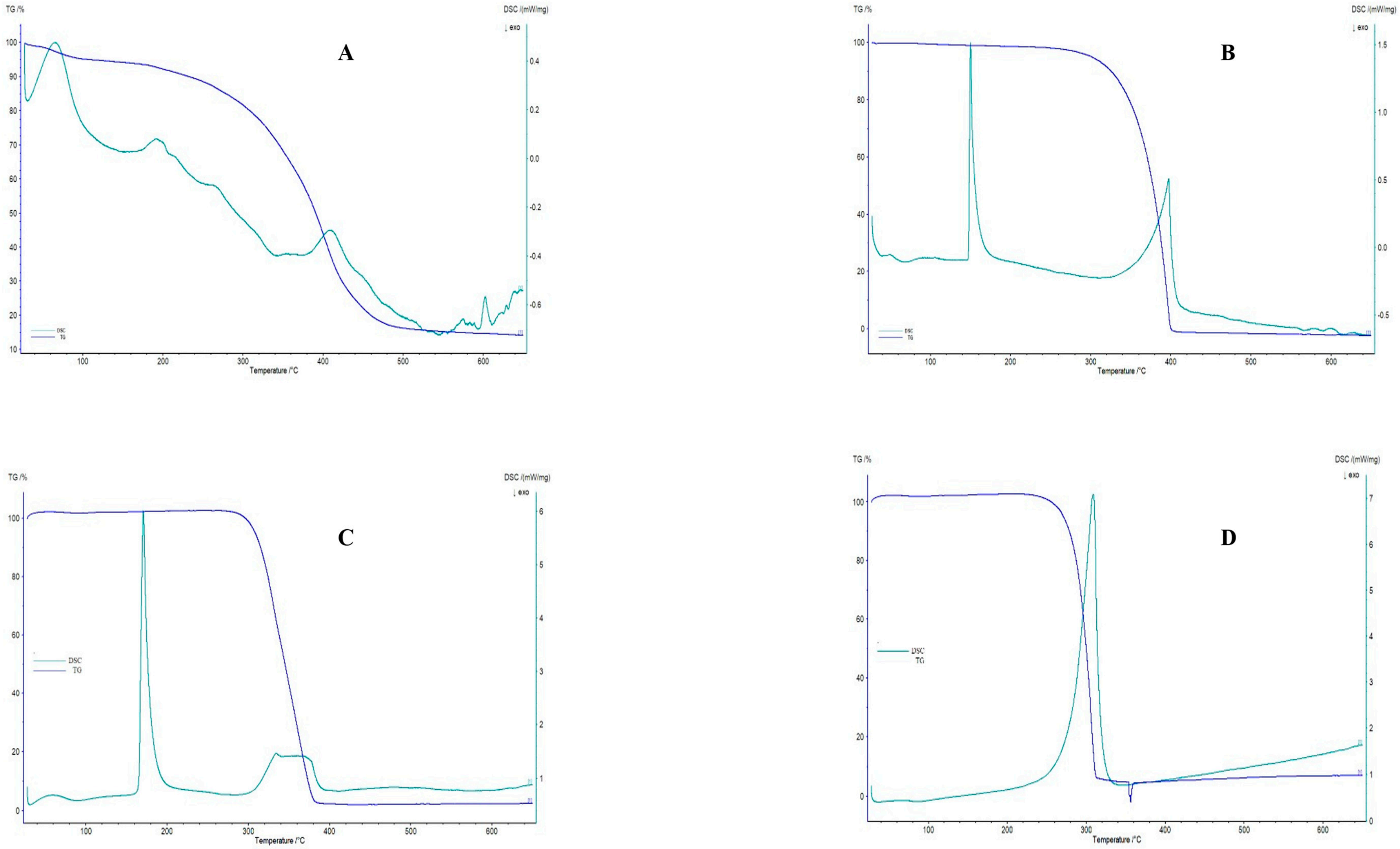

Figure S5. DSC/TGA thermograms. (**A**) Phosphatidylcholine, (**B**) Cholesterol, (**C**) D-mannitol, (**D**) L-leucine.

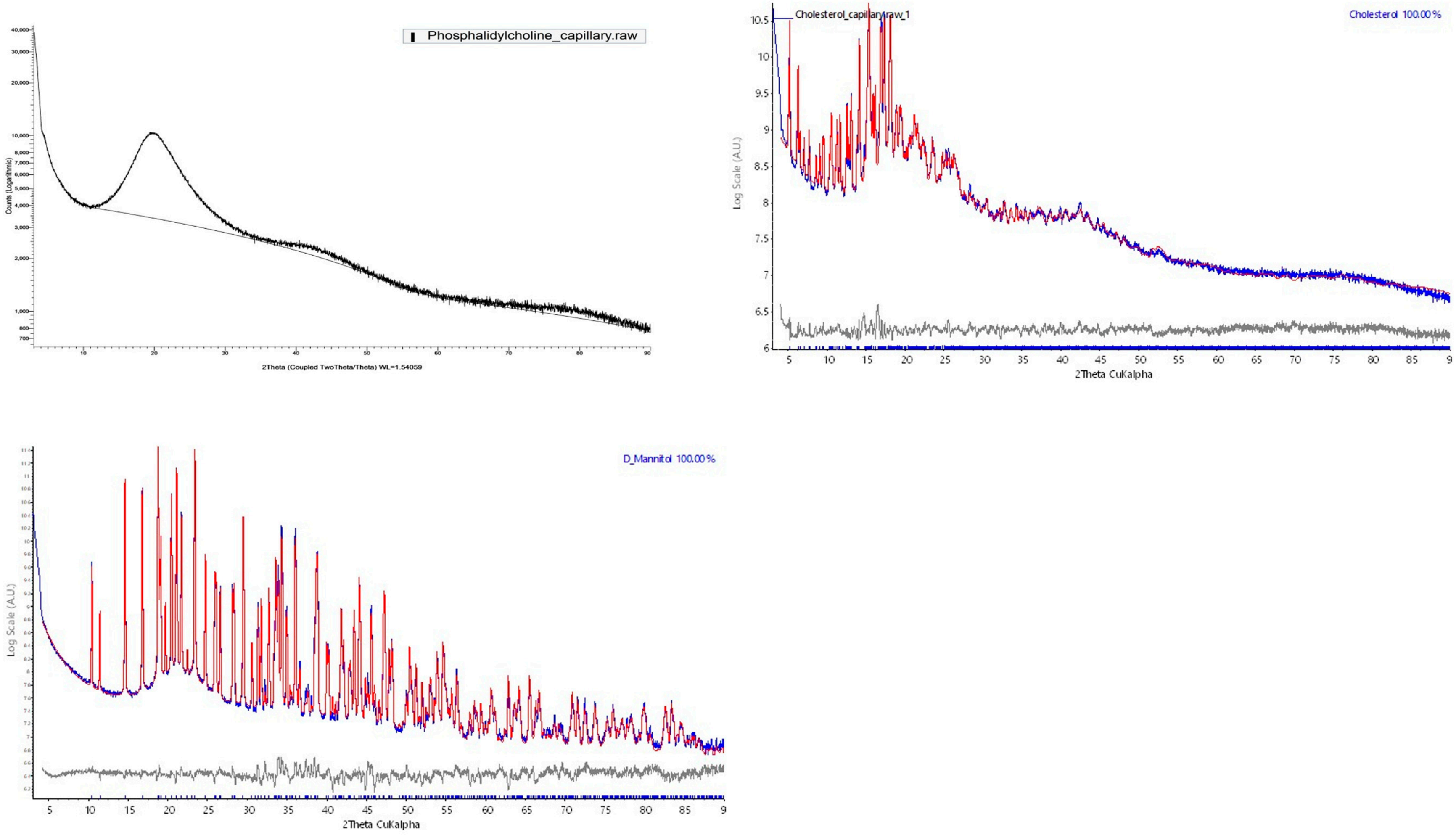

Figure S6. XRD patterns of Phosphatidylcholine, Cholesterol, and D-mannitol.
